# Supplementary material for: Clinically Relevant Oxygraphic Assay to Assess Mitochondrial Energy Metabolism in Acute Myeloid Leukemia Patients
Source: Cancers (Basel). 2021 Dec 17;13(24):6353. doi: 10.3390/cancers13246353 (PMC8699320; doi:10.3390/cancers13246353)
Supplement: Supplementary file 1 [file cancers-13-06353-s001.zip › cancers-1469453-supplementary proof final.pdf]

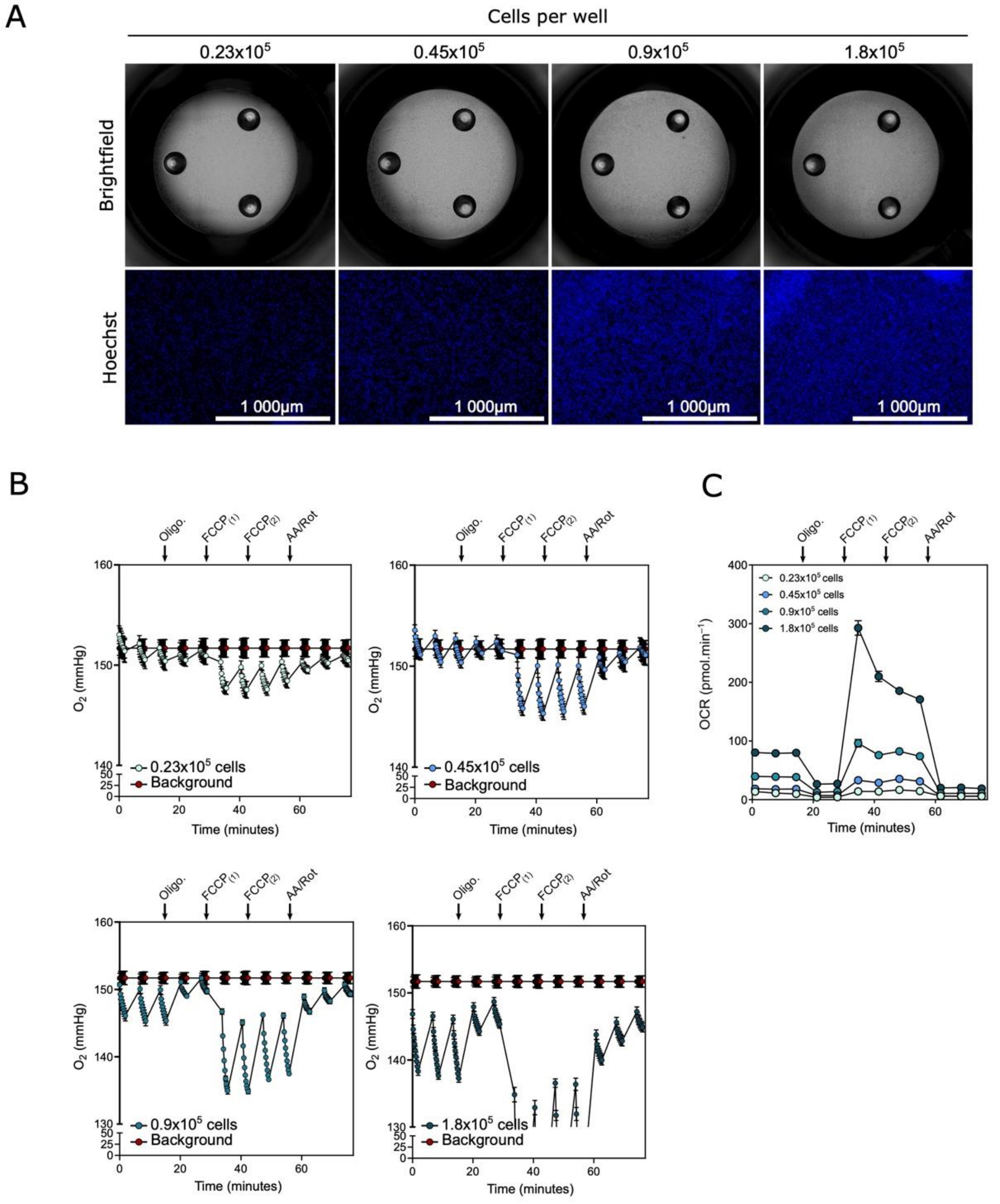

Supplementary Figure S1. Number of AML cells required to measure oxygen consumption with XFe96 Seahorse. (A) Top panel: Cellular imaging of AML blasts (0.23×10^5 to 1.8×10^5 cells). Images display the wells of the XFe96 cell plates obtained by bright field light microscopy. Middle panel: Nuclei were stained using a Hoechst probe and visualized with a Cytation I fluorescence cell imager using a DAPI filter (scale bar = 1000 µm). (B) Oxygen levels (mmHg) measured in the medium surrounding the AML blasts according to the number of cells per well. At the times indicated (see black arrows), the following drugs were injected: oligomycin A (Oligo; 2 µM), FCCP1 (1.1 µM), FCCP2 (2.2 µM), antimycin A and rotenone (AA/Rot; 1 µM each). Red dots represent the oxygen levels measured in the wells containing only medium (used for background correction). (C) Oxygen consumption rate (OCR; $pmol.min^{-1}$) of the blasts from AML patients according to the number of cells per well. Data are the means ± SEM (at least n = 3 wells per group). All experiments were performed with blasts freshly collected from the blood of patient #21.

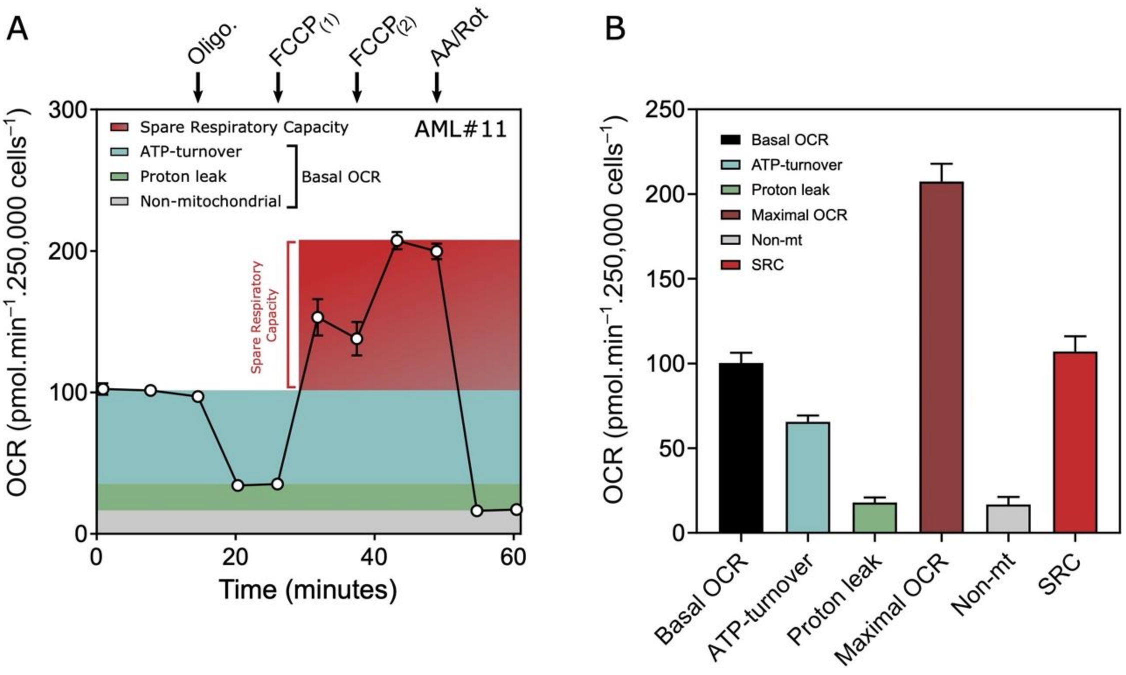

Supplementary Figure S2. (A) Typical OCR assessment profile of the blasts from AML patients. The oxygen consumption rate (OCR; pmol.min^{-1}.250,000. cells^{-1}) of the blasts from an AML patient. Data are the means ± SEM (at least n = 3 wells). Experiments were performed with blasts freshly collected from the blood of patient #11 and cultivated for 18 hr in full RPMI medium supplemented with cytokines. At the times indicated (see black arrows), the following drugs were injected: oligomycin A (Oligo; 2 µM), FCCP1 (1.1 µM), FCCP2 (2.2 µM), antimycin A and rotenone (AA/Rot; 1 µM each). (B) The basal OCR (blue + green + gray areas) of the blasts was determined between t0min to t15min. After exposure to oligomycin A from t20min to t26min, the OCR was related to the mitochondrial respiration used to generate ATP (ATP turnover, blue area). The remaining respiration, known as the proton leak (green area), is linked to oxygen-consuming mitochondrial processes independent of ATP production. Following exposure to FCCP (from t32min to t51min), oxygen consumption increased until maximal oxygen consumption was reached. The mitochondrial spare respiratory capacity (SRC; red area) was calculated by subtracting the FCCP-stimulated OCR (maximal OCR) from the basal OCR. Finally, protein complexes I and III of the respiratory chain were inhibited by a mixture of rotenone and antimycin A to determine the nonmitochondrial respiration (gray area) of the blasts (from t56min to t61min).

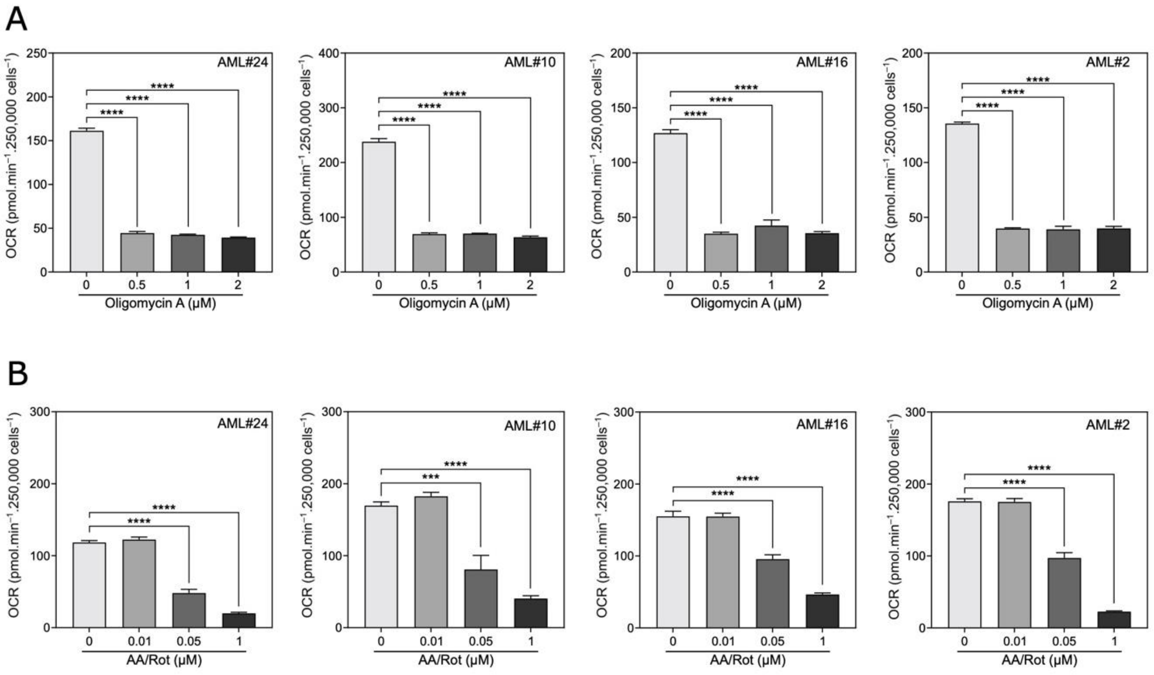

Supplementary Figure S3. Inhibition of the oxygen consumption rate in AML blasts after exposure to oligomycin A or antimycin A/rotenone. (A) Oxygen consumption rate (OCR; pmol.min^{-1}.250,000 cells^{-1}) of the blasts from AML patients after exposure to the indicated concentration of oligomycin A for 6 min. (B) Oxygen consumption rate (OCR; pmol.min^{-1}.250,000 cells^{-1}) of the blasts from AML patients after exposure to the indicated concentrations of rotenone and antimycin A for 6 min (same concentration for both inhibitors). Data are the means ± SEM (at least n = 3 wells per group). *$P<0.05$; **$P<0.01$; ***$P<0.001$; ****$P<0.0001$ compared to the control. Experiments were performed with the blasts from the patients indicated in the upper right corner of each histogram.

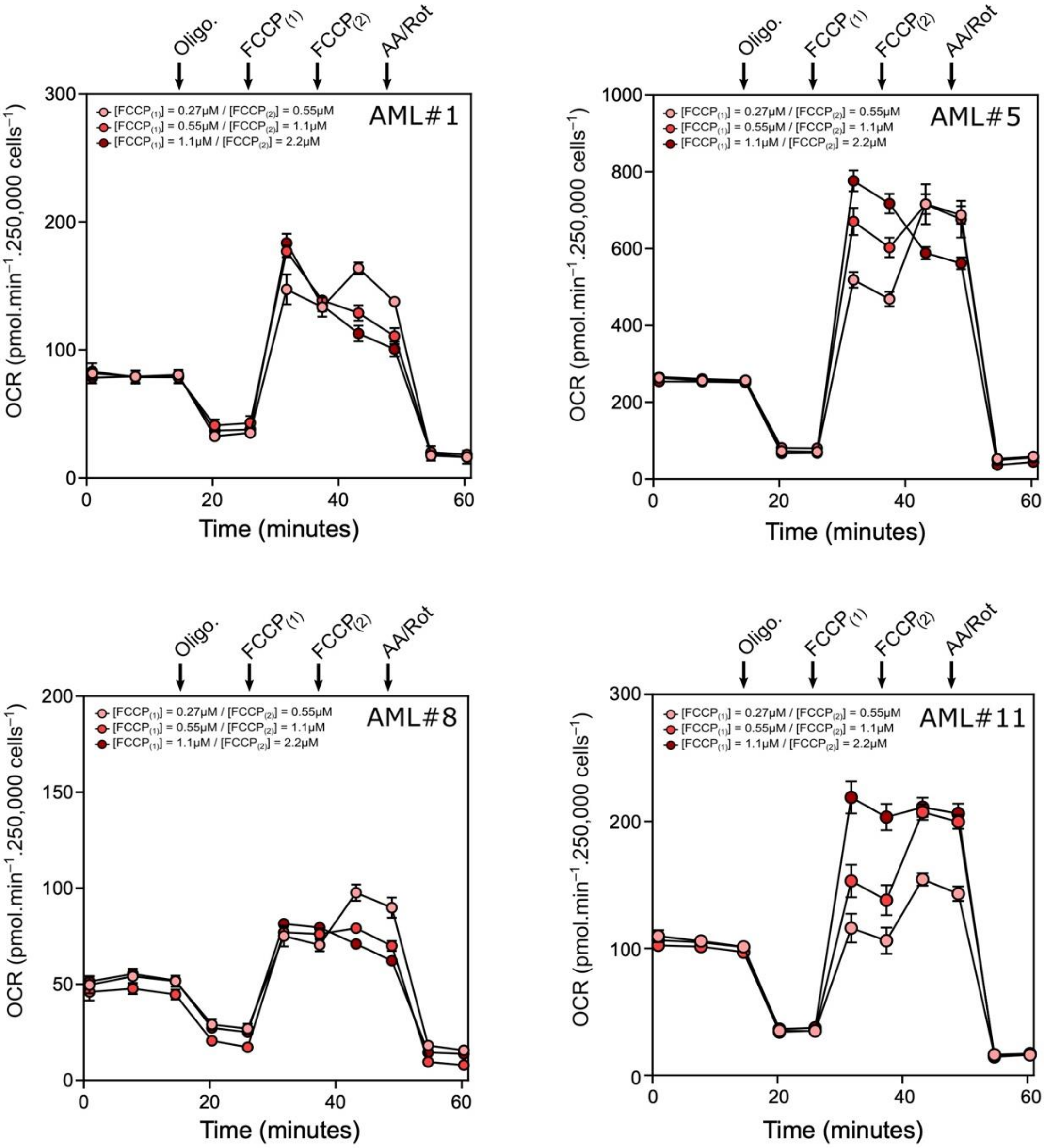

Supplementary Figure S4. Typical OCR assessment profile of the blasts exposed to increasing concentrations of FCCP. The oxygen consumption rates (OCRs; pmol.min^{-1}.250,000 cells^{-1}) of the blasts from AML patients were determined with XFe24 Seahorse (see Fig. 7B for template organization). Data are the means ± SEM (at least n = 3 wells). Experiments were performed with blasts freshly collected from the blood of patient #14 and cultivated for 18 hr in full RPMI medium supplemented with cytokines. At the times indicated (see black arrows), the following drugs were injected: oligomycin A (Oligo; 2 μM), FCCP1 (see indicated concentration), FCCP2 (see indicated concentration), antimycin A and rotenone (AA/Rot; 1 μM each).

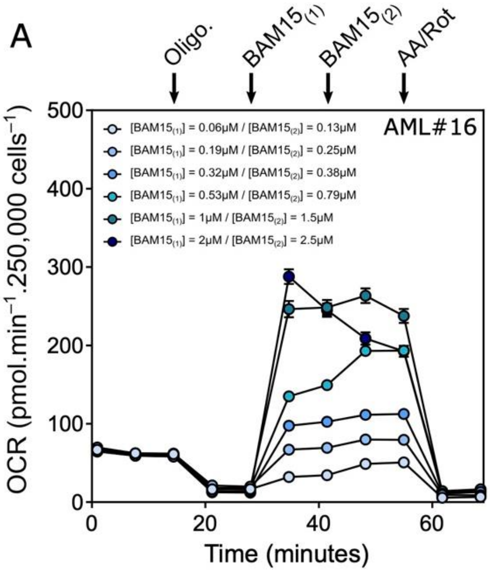

Supplementary Figure S5. Typical OCR assessment profile of the blasts exposed to increasing concentrations of BAM15. The oxygen consumption rates (OCRs; pmol.min^{-1}.250,000 cells^{-1}) of the blasts from AML patients were determined with XFe24 Seahorse. Data are the means ± SEM (at least n = 3 wells). Experiments were performed with blasts thawed from the blood of patient #16 and cultivated for 18 hr in full RPMI medium supplemented with cytokines. At the times indicated (see black arrows), the following drugs were injected: oligomycin A (Oligo; 2 µM), BAM15(1) (see indicated concentrations), BAM15(2) (see indicated concentrations), antimycin A and rotenone (AA/ Rot; 1 µM each).

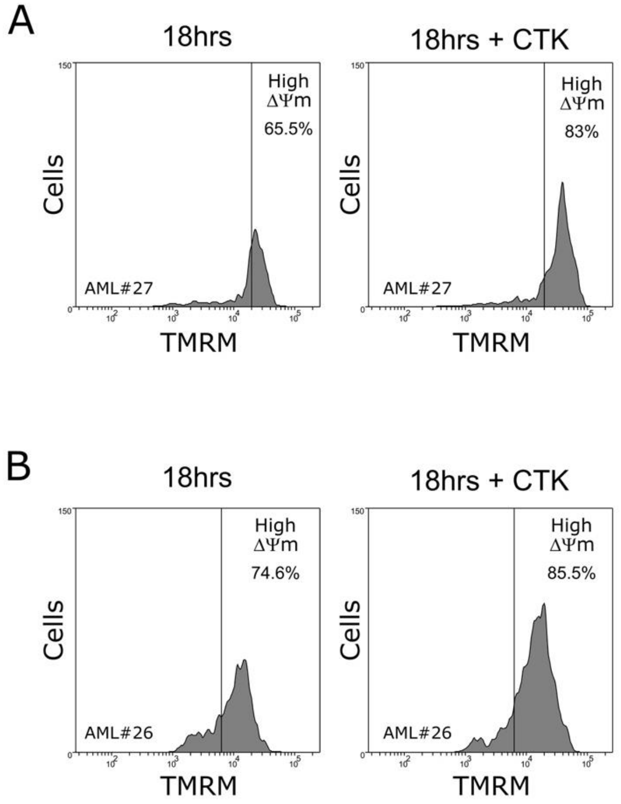

Supplementary Figure S6. Mitochondrial membrane potential in living blasts cultured for 18 hr (from patients #27 and #26). Thawed blasts from the indicated AML patients were cultured in full RPMI (for 18 hr) or in full RPMI supplemented with cytokines (18 hr + CTK). Viable blasts were identified under both conditions by flow cytometry following Annexin V-APC and SYTOX blue staining, and the percentages of blasts with a high mitochondrial membrane potential (ΔΨM) were determined by TMRM labeling (percentages are indicated in the top right corner of the cytometry profile). Cytofluorimetric profiles are representative of two biological replicates.

Supplementary Table S1. XFe96 Settings for mitochondrial OXPHOS measurement

Settings	Cycles	Mix	Wait	Measure	Total duration
Basal	3	2 min 20 s	2 min	2 min	19 min
Oligomycin (2μM)	2	2 min 20 s	2 min	2 min	12 min 40 s
$FCCP_1$ (0.27-1.1μM) or BAM15 (0.06-2μM)	2	2 min 20 s	2 min	2 min	12 min 40 s
$FCCP_2$ (0.55-2.2μM) or BAM15 (0.13-2.5μM)	2	2 min 20 s	2 min	2 min	12 min 40 s
Antimycin A + Rotenone (1μM each)	3	2 min 20 s	2 min	2 min	19 min
